# Microsatellite Polymorphisms Adjacent to the Oxytocin Receptor Gene in Domestic Cats: Association with Personality?

**DOI:** 10.3389/fpsyg.2017.02165

**Published:** 2017-12-18

**Authors:** Minori Arahori, Hitomi Chijiiwa, Saho Takagi, Benoit Bucher, Hideaki Abe, Miho Inoue-Murayama, Kazuo Fujita

**Affiliations:** ^1^Department of Psychology, Graduate School of Letters, Kyoto University, Kyoto, Japan; ^2^Japan Society for the Promotion of Science, Tokyo, Japan; ^3^Wildlife Research Center, Kyoto University, Kyoto, Japan; ^4^Wildlife Genome Collaborative Research Group, National Institute for Environmental Studies, Tsukuba, Japan

**Keywords:** domestic cat, microsatellite polymorphism, mongrel cat, oxytocin receptor gene, personality, purebred cat

## Abstract

A growing number of studies have explored the oxytocin system in humans and non-human animals, and some have found important genetic polymorphisms in the oxytocin receptor gene (*OXTR*) associated with the bonding system, social behaviors, and personality in several species. Although single nucleotide polymorphisms in *OXTR* have been well-examined in various species, microsatellites (or short tandem repeats) adjacent to *OXTR* have rarely been studied, despite some suggestions that microsatellite polymorphisms near genes might play a role in genetic transcription and translation. In this study, we surveyed microsatellites in the upstream, intron, and downstream regions of *OXTR* in domestic cats (*Felis catus*). We succeeded in amplifying 5 out of 10 regions, and recognized these five regions as polymorphic. We compared allele frequencies in these five regions between mongrel cats in Japan (*n* = 100) and cats of 10 pure breeds (*n* = 40). There were significant differences in allele frequencies between the two populations in all microsatellite regions. Additionally, the owners of mongrel cats answered a comprehensive personality questionnaire, and factor analysis extracted four factors (Openness, Friendliness, Roughness, and Neuroticism). We examined the association between the microsatellite genotypes, age, sex, neutering status, and personality scores. Compared to their counterparts, younger cats tended to score higher on Openness, male cats scored higher on Friendliness, and female and neutered cats scored higher on Roughness. When we divided the sample into three groups depending on the length of alleles, we found a marginally significant association between Friendliness and MS3. Additionally, we found a sex-mediated effect of genotypes in MS4 on Friendliness, resulting in different effects on females and males. Our findings that mongrel cats had longer alleles in MS3 and MS4 than purebred cats, and that those cats tended to score higher on Friendliness, supported the previous findings. However, future studies such as comparison between purebred cats with apparently different origin or personality are required to determine the association of genetic variants in the *OXTR* with personality.

## Introduction

Many studies focusing on the oxytocin system in humans have revealed that some genetic polymorphisms are associated with a large variety of individual differences in, for example, empathy (e.g., Rodrigues et al., 2009; Wu et al., 2012; Laursen et al., 2014), attachment anxiety (e.g., Chen and Johnson, 2012), prosociality (e.g., Shang et al., 2017), and pair-bonding behavior (e.g., Walum et al., 2012). In parallel, studies on non-human animals have explored genetic polymorphisms in the oxytocin receptor gene (*OXTR*) as a candidate gene related to the bonding system, social behaviors, and personality traits (e.g., Aspé-Sánchez et al., 2016). For *OXTR* in non-human primates, Staes et al. (2014) tried to find genetic differences between chimpanzees and bonobos at the locus of rs53576 in the intron 3 region of *OXTR*, which had been shown to be involved in many associations in humans (e.g., Rodrigues et al., 2009; Laursen et al., 2014); they expected this locus would contribute to species differences in empathy between chimpanzees and bonobos. Although they did not find polymorphism at this locus, they found novel polymorphisms near rs53576 in these two species. To date, these polymorphisms have not been shown to be associated with behavioral traits within species (chimpanzee; Staes et al., 2015); other studies have suggested the impact of the oxytocin system on primates’ bonding systems (Lee et al., 2011; Vargas-Pinilla et al., 2015). In fact, New World monkeys showed a new type of the oxytocin gene (*OXT*) (one amino acid was changed compared to the wild type) with co-evolved *OXTR* with positive selection, indicating an association with different systems in animals’ mating (e.g., monogamy). Concerning companion animals, Kis et al. (2014) reported that three single nucleotide polymorphisms (SNPs) in domestic dogs were associated with dogs’ responses to unfamiliar humans; however, Ottenheimer-Carrier et al.’s (2017) questionnaire study failed to provide further support to this finding in various breed groups, suggesting the possibility that gene-personality associations may reflect multiple factors, including breed, early experiences, and experimental contexts. Our previous study on domestic cats (*Felis catus*) (Arahori et al., 2016) reported an association between one SNP and a personality trait (Roughness). However, the molecular and functional mechanisms are still unclear.

Most studies referring to gene-behavior associations have focused on SNPs. However, in addition to SNPs, microsatellites (or short tandem repeats) adjacent to genes have been suggested to play a role in genetic transcription and translation (Sawaya et al., 2013). In particular, microsatellites near to or on the promoter region, such as TATA boxes (transcription start sites), GC-rich regions, and CAAT boxes within upstream regions could affect gene expression. In non-human animals, Oliva et al. (2016) compared microsatellites between wolves and dogs in the region close to *OXTR*, and found differences in the frequencies, but no differences in performance (scores) in an object choice task depending on length of alleles. Lonn et al. (2017) revealed that microsatellites in the 5′ regulatory region of *OXTR* and arginine vasopressin receptor gene 1a (*AVPR1A*) were associated with reproductive success demonstrated in field experiments and gene expression in the brains of bank voles. However, little research on *OXTR* has been conducted on animals, including domestic cats.

Domestic cats, which are common companion animals, are thought to have been domesticated almost 10,000 years ago in the Near East (Driscoll et al., 2007). Their gene-personality associations have been under-researched—our previous research is an exception (Arahori et al., 2016). These associations are providing an important clue to understand how African wild cats (the ancestors of domestic cats) adapted to humans and human societies by comparing personality-related genes found in domestic cats from an evolutionary developmental biology point of view, and infer their pathway from wildcats to pets. The association would also be important for cats’ welfare because personality-related genes could suggest matching between potential owners and cats.

In addition to the early phase of cat domestication, many cat breeds have more recently been created through various degrees of artificial selective pressure, according to humans’ preferences (mainly in relation to appearance, although sometimes also temperament—e.g., the Ragdoll with its high placidity; Bradshaw et al., 1999). Some previous studies have described breed-specific personality traits as reported by veterinarians and owners in response to questionnaires. Takeuchi and Mori (2009) reported that Japanese domestic cats (mongrel cats) and American Shorthair cats ranked higher on friendliness, playfulness, demand for affection, and novelty seeking than 10 other pure breeds examined in the study, whereas Chinchilla cats were ranked highest on aggression to humans and cats, timidity, and nervousness. Wilhelmy et al. (2016) examined the links between appearance (e.g., coat color) and personality, but concluded that most individual differences stemmed from breed differences. In sum, the artificial selection of cat breeds may have influenced breed-specific personality, suggesting that some heritable genes would be linked to personality. Therefore, examining differences in terms of *OXTR* between mongrel cats and purebred cats might be one of the keys to reveal the effects of *OXTR* on the personality of cats.

In this study, we explored the microsatellites adjacent to *OXTR* in cats. First, we compared allele frequencies in microsatellites between mongrel cats and purebred cats because we expected that *OXTR* in purebred cats under human selective pressures would be different genetically from that in mongrel cats. Possibly, purebred cats must have been selectively bred for appearance by humans, although it is unclear that such selection could also influence behavior and personality. On the other hand, mongrel cats are not generally selected by humans. In fact, in 2016 in Japan, 57.1% of cat owners reported that they adopted free-roaming cats as their pet(s), either by themselves or via organizations (e.g., animal shelters) (Japan Pet Food Association, 2016), suggesting free-roaming cats’ histories were much different from purebred cats in Japan. Therefore, we expected that sequences in *OXTR*, the candidate gene related to social behavior, would differ between mongrel cats (free-roaming for several generations) and purebred cats (having undergone artificial selection). Second, we examined the association with personality scores in mongrel cats to ascertain the effects of *OXTR* polymorphisms on personality. Only mongrel cats were examined due to the difficulty of finding single breed subjects with little or no kinship. We considered not only *OXTR* genotypes and the effects of age, sex, and neutering on cat personality, but also sex-mediated effects connected with *OXTR*, as suggested in human studies (e.g., Stankova et al., 2012).

## Microsatellites in *OXTR* and the Differences Between Mongrel Cats and Purebred Cats

### Materials and Methods

#### Subjects

We included 100 cats (54 males, 46 females; mean age = 60.95 months; standard deviation = 52.05 months) in Japan. All cats were mongrels born in Japan, suggesting that their ancestors could not be identified as purebred according to the owners. According to the owners, 35 cats were free-roaming cats that had been taken home by the owners themselves, 23 were adopted by the owners via their acquaintances, 17 were adopted by owners via animal shelters or volunteers, 3 comprised siblings of cats that owners already kept, and we were unable to obtain answers from 22 cat owners. A total of 88 cats were neutered (48 males, 40 females), while 12 cats were not (6 males, 6 females); 85 were household pets (48 males, 37 females) and the remaining 15 were residents in “cat cafés” (6 males, 9 females), where guests interact with them, resulting in extensive contact with people almost every day. All cats had one or two owners (caretakers). Based on owners’ reports, none of the cats in the sample were genetically related. We included more than one cat belonging to the same owner if these cats showed different haplotypes in examined microsatellites.

To compare allele frequencies between mongrel and purebred cats, we selected an additional 40 genetically unrelated cats of 10 different typical cat breeds (4 cats each: 2 males, 2 females; Abyssinian, American Curl, American Shorthair, Scottish Fold, Somali, Persian, Himalayan, Chinchilla Persian, Maine Coon, and Russian Blue). These cats were used in a previous study (Kato et al., 2007; in Japanese), and they were offered to us via veterinary hospitals from cat owners (Veterinarians confirmed their breeds, and the owners offered information about their kinships).

#### Microsatellite Searching

**FIGURE 1:**
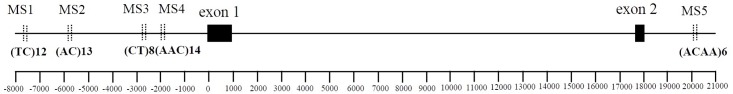
Conceptual diagram of *OXTR* and the locus of microsatellites in cats.

From the cat genome sequence (GenBank assembly accession: GCA_000181335.3) we searched for microsatellites in the upstream region (8 kb) of exon 1, the intron region between exons 1 and 2, and the downstream region (3 kb) of exon 2 in *OXTR* using WebSat software (Martins et al., 2009). We found 10 regions including microsatellites (4 in the upstream region, 5 in the intron region, and 1 in the downstream region; **Figure 1**); however, we analyzed only 5 (MS1, 2, 3, 4, and 5) of these 10 regions. We could not amplify those in the intron region when considering polymerase chain reaction (PCR) conditions because there were probably numerous mutations in this region.

#### Sample Collection and Genotyping

For mongrel cats, we collected buccal cell samples after obtaining permission from their owners. Buccal cell samples were collected using cotton swabs (JCB INDUSTRY LIMITED, Ginza, Chuou-ku, Japan) in 2 ml of 0.9% saline solution, then 9 ml of 99.5% ethanol was added for preservation, and stored at 4°C until DNA extraction.

DNA was extracted from the buccal cells of cats using a QIAamp blood and tissue kit (QIAGEN, Valencia, CA, United States). Five microsatellite regions were amplified by PCR with a 10 μl mixture for each sample, containing 5 μl of Multiplex PCR kit (QIAGEN, Valencia, CA, United States), 1 μl template DNA, 3.5 μl H_2_O, and 0.05 μl for each forward and reverse primer (see **Table 1** for primer information). The PCR conditions consisted of 95°C preheating for 15 min, 35 cycling at 94°C for 30 s, 60°C for 30 s, 72°C for 30 s, and 60°C for 30 min as the last extension. Subsequently, we sequenced PCR products using the Applied Biosystems *3130xl* Genetic Analyzer (Applied Biosystems, Foster City, CA, United States). The sizes of PCR products were genotyped using the GENESCAN software package (PerkinElmer, Foster City, CA, United States). We checked peaks (the sizes of PCR products), and repeated the analysis if the peaks were unclear to determine.

**Table 1:** Primer sequence used for this study.

MS	Primer (5′–3′)	Fluorescent dyes	Repeat unit	Size (bps)	No. of alleles
1	CTCCTGAATGTGGGTGGGAC	NED	(TC)12	217	4
	TCAGAGCGCCTGTGAATGAG
2	AAAGGTGAAGCAGAAAGTGGAG	FAM	(AC)13	396	7
	ACCTCCAGTGAAAAGTGACAGA
3	GGTGGCTCAGTCAGTGACTC	HEX	(CT)8	235	2
	GGTGATGTGGGGCTTAGCAT
4	TGGTTTTCCCTGTCTTCATTCT	HEX	(AAC)14	365	9
	TTTGTTCCTATTCCCATTCCTG
5	CCTGCATTTGGGGTAGAGATTA	FAM	(ACAA)6	308	2
	CACCAGCAACGTATGAGAGTTC


#### Statistics

We assessed linkage disequilibrium among all microsatellites. We used Fisher’s exact test to assess the differences in allele frequencies between mongrel and purebred cats. We used R (v. 3.4.2) for all statistical analyses (R Core Team, 2017) using the package: *genepop* (Rousset, 2008).

### Results

#### Linkage Disequilibrium among Five Microsatellite Loci

All five regions were polymorphic in both (mongrel and purebred) cat groups. For mongrel cats, the test for genotypic linkage disequilibrium (LD) revealed non-random associations among microsatellite loci (*p* < 0.01) other than MS4 and MS5 (*p* = 0.694).

#### Allele Frequency Differences between Mongrel and Purebred Cats

Overall, alleles of mongrel cats in MS1, MS2, MS3, and MS4 were longer than in purebred cats, but the difference in MS5 was reversed (**Table 2**). In MS2 and MS4, mongrels had more alleles than purebred cats did. Fisher’s exact test revealed significant differences between mongrels and purebred cats in all alleles (MS1: *p* < 0.0001; MS2: *p* < 0.01; MS3: *p* < 0.0001; MS4: *p* < 0.0001; and MS5: *p* < 0.001).

**Table 2:** Allele frequencies observed in Japanese mongrel cats and purebred cats.

Locus	*He*	Allele frequencies								
**MS1**		211	215	217	219					
Mongrel	0.61	0.14	0.195	0.57	0.095					
Pure breed	0.74	0.138	0.288	0.3	0.275					
**MS2**		392	396	406	408	410	412	414		
Mongrel	0.81	0.08	0.015	0.08	0.13	0.2	0.2	0.295		
Pure breed	0.81	0.288	0	0.063	0.125	0.125	0.213	0.188		
**MS3**		235	237							
Mongrel	0.44	0.33	0.67							
Pure breed	0.49	0.6	0.4							
**MS4**		356	359	362	365	368	371	374	377	380
Mongrel	0.78	0	0.005	0.235	0.325	0.185	0.15	0.08	0.005	0.015
Pure breed	0.58	0.013	0.125	0.15	0.613	0.1	0	0	0	0
**MS5**		304	308							
Mongrel	0.28	0.83	0.17							
Pure breed	0.47	0.625	0.375							


**He* represents expected heterozygosity in each population. The numbers next to each population mean allele frequencies in each allele. Underlined numbers indicate the alleles divided into *L*.*

## Gene-Personality Associations in Mongrel Cats

### Materials and Methods

#### Subjects and DNA Collection

We used the same mongrel cat subjects (*n* = 100) as those used to assess differences between mongrel cats and purebred cats in microsatellites adjacent to *OXTR* (see section “Microsatellites in *OXTR* and the Differences between Mongrel Cats and Purebred Cats”).

#### Rating of Cat Personality

We used a personality questionnaire developed for Japanese Akita dogs (Konno et al., 2011), which was used in our previous study with cats (Arahori et al., 2016). It consists of 30 questions measured on a six-point scale. The caretaker of each mongrel cat (including “cat café” owners) completed the questionnaire. Additionally, we included data from the cat sample (only mongrel cats) in Arahori et al. (2016) (32 males, 25 females; mean age = 60.0 months; standard deviation = 50.55 months), in which test–retest reliability was satisfactory.

#### Statistics

First, we inspected the scree plot by running a parallel analysis to determine the number of factors. Next, we performed a factor analysis with maximum likelihood estimation and promax rotation to reveal the cats’ personality structure, and extracted items with factor loadings greater than |0.5| . We calculated factor scores using the regression method.

For gene-personality association, we divided samples into three groups by genotype (*L/L*, *S/L*, *S/S*; see **Table 2** for grouping) to make the sample size and number of alleles in *S* and *L* of mongrel cats as equal as possible. After dividing, pairwise LD measurements (D′) were calculated to assess the linkage of each microsatellite. We used a generalized linear model (GLM) to examine the association between factor scores as the response variable and independent variables; age (months), neutering status, sex, target genotype (*L/L*, *S/L, S/S*) and interaction of sex and genotypes were included as fixed effects. We visually checked the residual plots and normal Q–Q plots to confirm our assumption (normal distribution and homogeneity of variance) for each model. We used R (v. 3.4.2) for all statistical analyses (R Core Team, 2017), as well as the packages: *psych* (Revelle, 2015), *genetics* (Warnes and Warnes, 2007), *LDheatmap* (Shin et al., 2006), and *car* (Fox and Weisberg, 2011).

### Results

#### Factor Analysis

We excluded questionnaire data (gene-behavior association test) of two owners due to missing data. Both the scree plot and a parallel analysis indicated four factors, and a factor analysis revealed the cats’ personality structure based on the questionnaire. The items in each factor were almost identical to those in Arahori et al. (2016). Therefore, we named these four factors Openness, Friendliness, Roughness, and Neuroticism (**Table 3**). The 23 items had loadings over |0.5| on each factor except for the item “adaptable,” which had a negative loading on Neuroticism but a positive loading on Friendliness (**Table 3**; underlined values). The Cronbach’s alpha for each factor was acceptable (**Table 3**).

**Table 3:** Factor loadings for the questionnaire.

	Openness	Friendliness	Roughness	Neuroticism
Playful	**0.923**	-0.04	-0.273	-0.088
Active	**0.807**	-0.139	-0.208	-0.244
Curious	**0.722**	0.231	0.131	-0.072
Inquisitive	**0.692**	0.096	0.106	-0.013
Inventive	**0.633**	0.037	0.012	0.051
Focused	**0.629**	0.223	-0.128	0.147
Mischievous	**0.523**	-0.106	0.168	-0.023
Vigilant	-0.119	-0.015	0.06	**0.755**
Fearful	-0.018	-0.065	0.113	**0.75**
Attentive	-0.137	0.054	0.008	**0.743**
Nervous	-0.116	0.125	0.167	**0.709**
Timid	0.046	-0.24	-0.068	**0.683**
Anxious	0.06	-0.18	0.117	**0.554**
Irritable	-0.131	-0.007	**0.889**	0.007
Moody	-0.15	0.022	**0.807**	0.148
Defiant	0.014	0.068	**0.797**	0.099
Dominant	-0.018	0.013	**0.782**	0.004
Aggressive	-0.167	-0.086	**0.576**	-0.066
Gentle	0.012	**0.745**	-0.184	0.088
Sociable	0.018	**0.725**	0.091	-0.235
Calm	-0.016	**0.671**	-0.033	-0.32
Friendly	0.056	**0.656**	0.074	-0.469
Adaptable	0.02	**0.632**	0.143	**–0.555**
Distractible	-0.136	-0.142	0.373	-0.295
Impulsive	0.295	-0.14	0.381	-0.058
Excitable	0.382	-0.134	0.44	0.062
Restless	0.304	-0.12	0.156	0.098
Quitting	-0.182	0.053	0.316	-0.196
Affectionate	0.143	0.363	0.053	0.059
Cautious	-0.005	0.382	-0.02	0.296
Variance explained (%)	13.9	10.8	13	14.3
Cronbach’s alpha	0.85	0.86	0.88	0.87


*The leftmost row represents all items used in this study. Cronbach’s alpha is shown in the bottom. The underlined values mean the item which showed both positive and negative loadings. The bold numbers indicate factor loadings of items included in each factor.*

#### Linkage of Genotypes (after Dividing into *L* and *S*)

**FIGURE 2:**
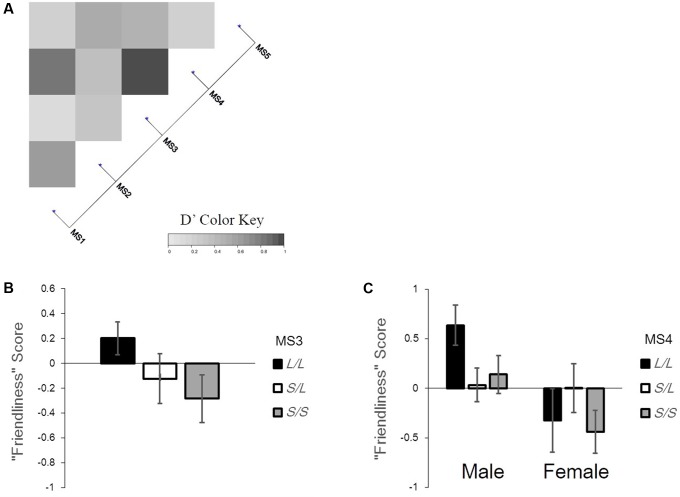
The linkage of microsatellites grouped for gene-personality analysis and the gene-personality association found in this study. **(A)** Pairwise LD measurements and the heatmap plot of five microsatellite grouping. **(B)** The association between the genotypes of MS3 and “Friendliness” scores. **(C)** The association between sex, the genotypes of MS4 and “Friendliness” scores. The error bars represent standard errors.

We plotted the correlation of genotypes on the heatmap using pairwise LD measurements (D′; **Figure 2A**). When dividing each allele into two groups (*S* and *L*) based on the sample size and number of alleles, the test showed that seven combinations out of 10 were in significant linkage disequilibrium (For MS2–MS4: *p* < 0.01; For MS1–MS2, MS1–MS4, MS2–MS3, MS2–MS5, MS3–MS4, and MS3–MS5: *p* < 0.001). There was no pair showing a complete linkage.

#### Association between Independent Variables and Personality Scores

We checked our assumptions to see the residual plots and normal Q–Q plots (**Supplementary Figures S1**, **S2**) as satisfactory. When grouping into three groups (*L/L*, *S/L, S/S*), a GLM analysis (see **Supplementary Table S1** for all results) revealed a significant difference in Openness according to age, with older cats scoring lower than younger cats (β = -0.008, standard error (SE) = 0.002, 95% Wald confidence interval (CI) = [-0.012, -0.003], χ^2^(1) = 12.558, *p* < 0.001). The effect of sex was significant on Friendliness, with females being less friendly than males (β = -2.251, *SE* = 1.333, 95% CI = [-4.863, 0.361], χ^2^(1) = 5.365, *p* = 0.021). MS3’s effect on Friendliness was marginally significant; cats with *S/S* alleles tended to score lower than cats with *L/L* and *S/L* alleles (**Figure 2B**; β = -1.227, *SE* = 0.595, 95% CI = [-2.393, -0.062], χ^2^(2) = 4.963, *p* = 0.084). Additionally, the interaction effect of sex × genotype in MS4 was significant (female × *S/L*: β = 1.784, *SE* = 0.696, 95% CI = [0.419, 3.148], χ^2^(2) = 8.308, *p* = 0.016; **Figure 2C**). Among males, the Friendliness score was higher in *L/L* groups than in other groups. On the other hand, among females, the Friendliness score was higher in *S/L* groups than in other groups. Sex and neutering effects were significant on Roughness, with females and neutered cats scoring higher than males and intact (not neutered) cats (sex: β = 2.266, *SE* = 1.344, 95% CI = [-0.368, 4.899], χ^2^(1) = 6.885, *p* = 0.009; neutering: β = 0.798, *SE* = 0.34, 95% CI = [0.131, 1.465], χ^2^(1) = 5.498, *p* = 0.019). We could not find any significant effect on the Neuroticism score.

## Discussion

This is the first study to survey microsatellites in the adjacent region of *OXTR* in cats. Microsatellites in the adjacent region of *OXTR* have been identified as important candidate genes related to social behavior in various species. We compared (1) allele frequencies between mongrel cats and purebred cats, and (2) examined the association between genotype and personality scores in mongrel cats.

First, the allele frequencies in five microsatellites were markedly different between mongrel and purebred cats. However, the test revealed significant linkage disequilibrium among almost all of the microsatellite loci, perhaps because they were located in the same gene.

Second, we analyzed the association between personality scores and the length of microsatellites in mongrel cats, and one significant and one marginal significant association were found with genotypes; the length of MS3 alleles and interaction of MS4 alleles and sex were associated with Friendliness. Taken together with the first finding, mongrel cats tended to have longer alleles in MS3 and MS4 than purebred cats, and those with longer alleles scored higher on Friendliness (**Figures 2B,C**). Takeuchi and Mori (2009) reported that Friendliness in Japanese domestic cats (mongrel cats) scored higher than in any other purebred cats examined. Our reports were consistent with their study; however, the functional reasons for the association for MS3 and MS4 were beyond the scope of our study. Because MS3 and MS4 were highly genetically correlated when dividing alleles into *L* and *S* (**Figure 2A**) and the locations on *OXTR* were near (**Figure 1**), they might have shown similar effects. We also found that male cats scored higher on Friendliness than did female cats, and their scores changed depending on the length of MS4 alleles. In humans, some studies have reported sex-mediated effects of genotypes in terms of *OXTR* (e.g., Stankova et al., 2012), and our previous study also found sex × neutering × *OXTR* genotype effects on Roughness scores in cats (Arahori et al., 2016).

Our study had several limitations. First, our purebred samples were mainly from eastern populations, and Persian, Chinchilla, and Himalayan have a similar origin (Alhaddad et al., 2013; The Cat Fanciers’ Association, 2017). Moreover, our sample size was too small for comparison among purebred cats. For future approaches, it would be recommended to carefully select 2 or 3 cat breeds using large sample size of each, with unique and easily comparable origins and selective pressures to reveal genetic differences related to their personality. As potential candidates one could think of using the Bengal breed (Gershony et al., 2014) for example, which is a hybrid of wildcats and domestic cats, or other carefully selected breeds that have already been revealed to differ in personality from mongrel cats. Lastly, we must note that GLM analysis was conducted after recognizing the high correlation (genetic linkage) among alleles/genotypes with possibilities of multicollinearity because they were positioned within the same gene.

Future studies have the potential to study other personality-related genes in cats and other felid species in terms of microsatellites. For example, *AVPR1A* is an important candidate gene related to social behavior in animals. In primates and prairie voles, microsatellites near (in the regulatory region of) *AVPR1A* are known to be related to mating systems, social organization, and sexual preferences (e.g., primates: Rosso et al., 2008; prairie voles: Castelli et al., 2011), as a result of comparing closely related species with different social systems. Only lions, cheetahs, and domestic cats are considered “social” felid species (Bradshaw, 2013), and this candidate-gene approach could reveal the *AVPR1A* effect.

## Conclusion

Our study has shown polymorphisms in microsatellites in domestic cats. Associations in mongrel cats and differences in allele frequencies with purebred cats showed consistency with previous findings (Takeuchi and Mori, 2009). However, the role of microsatellites in these non-coding regions is still unclear and further research is therefore necessary, using different carefully selected breeds of cats.

## Ethics Statement

This study adhered to the ethical guidelines of Kyoto University, and was approved by the Animal Experiments Committee of the Graduate School of Letters of Kyoto University (Approval reference number: 17-11).

## Author Contributions

MA designed this study, collected DNA sample and questionnaires, conducted genotyping, analyzed data, and drafted the manuscript. HA designed primers for genotyping. HC, ST, and BB contributed to data collection. MI-M and KF provided critical discussion regarding the analyses and the manuscript.

## Conflict of Interest Statement

The authors declare that the research was conducted in the absence of any commercial or financial relationships that could be construed as a potential conflict of interest.

## Supplementary Material

FIGURE S1 Residual plots were shown for checking visually the assumption for normality of residuals. **(a)** Openness, **(b)** Friendliness, **(c)** Roughness, and **(d)** Neuroticism.

FIGURE S2 Normal Q–Q plots were shown for checking visually homogeneity of variance. **(a)** Openness, **(b)** Friendliness, **(c)** Roughness, and **(d)** Neuroticism.

TABLE S1 In each sheet, the GLM results for Openness, Friendliness, Roughness and Neuroticism were shown.
