# Supplementary figures and images for: Microsatellite Polymorphisms Adjacent to the Oxytocin Receptor Gene in Domestic Cats: Association with Personality?
Source: Front Psychol. 2017 Dec 18;8:2165. doi: 10.3389/fpsyg.2017.02165 (PMC5741686; doi:10.3389/fpsyg.2017.02165)

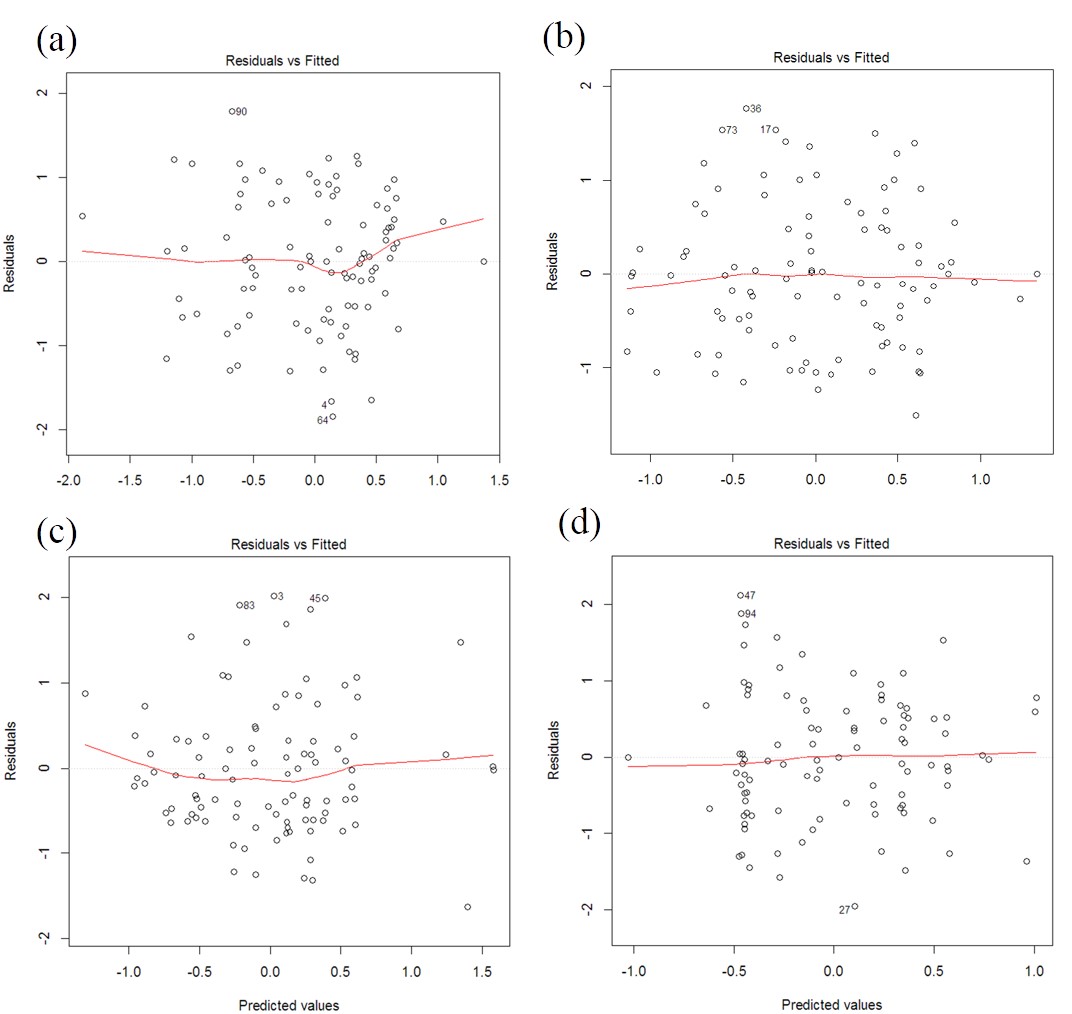

Supplement: FIGURE S1 — Residual plots were shown for checking visually the assumption for normality of residuals. (a) Openness, (b) Friendliness, (c) Roughness, and (d) Neuroticism. [file Image_1.JPEG]

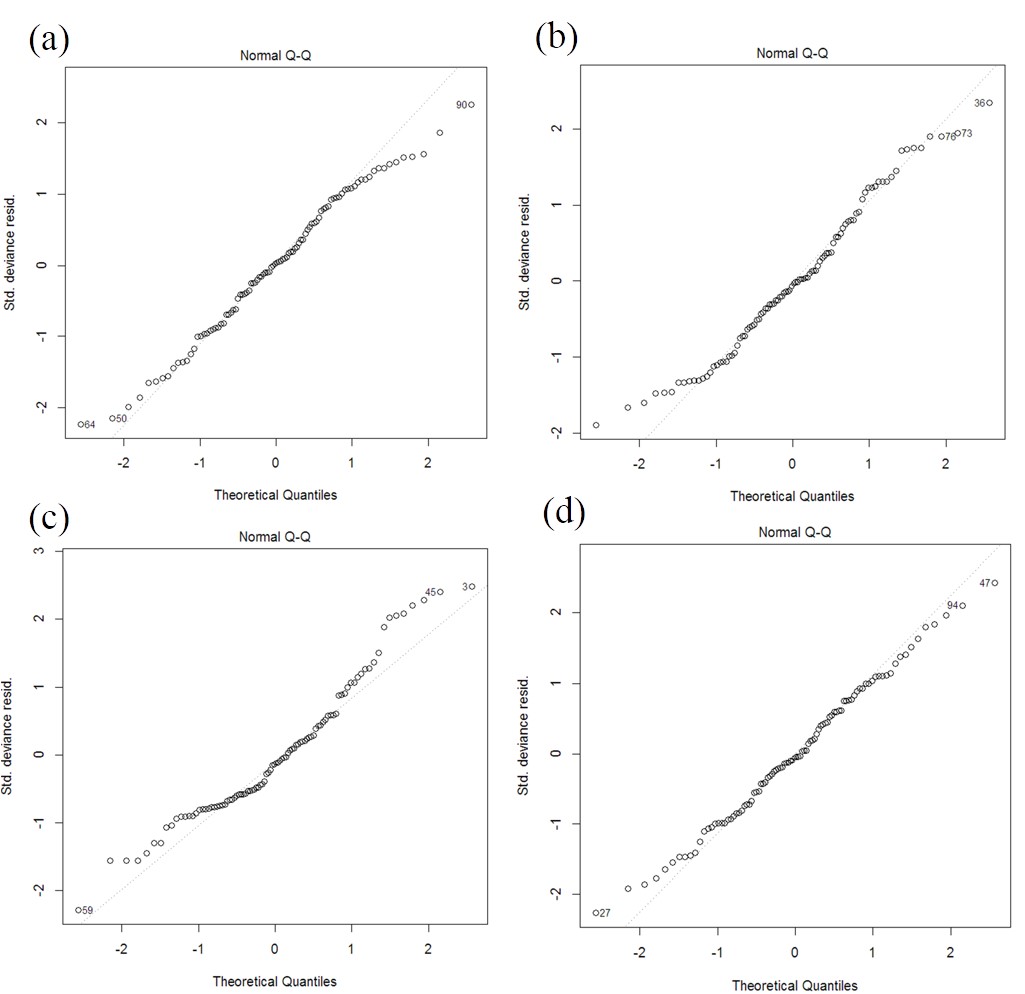

Supplement: FIGURE S2 — Normal Q–Q plots were shown for checking visually homogeneity of variance. (a) Openness, (b) Friendliness, (c) Roughness, and (d) Neuroticism. [file Image_2.JPEG]
